# Uremic Sarcopenia and Its Possible Nutritional Approach

**DOI:** 10.3390/nu13010147

**Published:** 2021-01-04

**Authors:** Annalisa Noce, Giulia Marrone, Eleonora Ottaviani, Cristina Guerriero, Francesca Di Daniele, Anna Pietroboni Zaitseva, Nicola Di Daniele

**Affiliations:** 1 UOC of Internal Medicine-Center of Hypertension and Nephrology Unit, Department of Systems Medicine, University of Rome Tor Vergata, Via Montpellier 1, 00133 Rome, Italy; e.ottaviani@hotmail.it (E.O.); cristinaguerriero@hotmail.it (C.G.); francesca.didaniele@gmail.com (F.D.D.); annapietroboni@icloud.com (A.P.Z.); didaniele@med.uniroma2.it (N.D.D.); 2 PhD School of Applied Medical, Surgical Sciences, University of Rome Tor Vergata, Via Montpellier 1, 00133 Rome, Italy

**Keywords:** uremic sarcopenia, physical activity, hemodialysis, nutritional supplementation, intra-dialytic parenteral nutrition, ω-3 polyunsaturated fatty acids

## Abstract

Uremic sarcopenia is a frequent condition present in chronic kidney disease (CKD) patients and is characterized by reduced muscle mass, muscle strength and physical performance. Uremic sarcopenia is related to an increased risk of hospitalization and all-causes mortality. This pathological condition is caused not only by advanced age but also by others factors typical of CKD patients such as metabolic acidosis, hemodialysis therapy, low-grade inflammatory status and inadequate protein-energy intake. Currently, treatments available to ameliorate uremic sarcopenia include nutritional therapy (oral nutritional supplement, inter/intradialytic parenteral nutrition, enteral nutrition, high protein and fiber diet and percutaneous endoscopic gastrectomy) and a personalized program of physical activity. The aim of this review is to analyze the possible benefits induced by nutritional therapy alone or in combination with a personalized program of physical activity, on onset and/or progression of uremic sarcopenia.

## 1. Introduction

“Sarcopenia” is a term derived from the Greek “sarx”—meat and “penia”—loss, and it was first coined in 1988 by Irwin Rosenberg to describe the modifications that occur in the muscles during aging [1]. Progressive and generalized loss of muscle strength and muscle mass (MM) is also a frequent complication in patients with chronic kidney disease (CKD), especially in end-stage renal disease (ESRD) [2,3,4]. The mechanisms involved are many and not yet fully clarified, but it is certain that they all converge towards a final process of increasing protein degradation and reducing protein synthesis, leading to a negative nitrogen balance [5]. Muscle is one of the most represented tissues in the human body. Skeletal muscles are the predominant component, while other types such as cardiac and smooth musculature are less represented. Skeletal muscle is mainly composed of proteins and it represents the “best indicator” of overall protein shift [6]. The reduction of muscle strength and mass, especially skeletal, is associated with a worsening quality of life (Qol), an increased vulnerability to adverse events such as falls, loss of personal autonomy and ultimately increased hospitalization and mortality [7].

In 1931 the British neurologist Critchley Macdonald was the first, in the scientific literature, to correlate aging with the tendency to loss of skeletal MM [7]. Since then, several studies have been carried out to deepen the qualitative and quantitative changes that occur physiologically in the MM and fat mass with aging [8].

Quantitative changes consist in the reduction of MM and volume, while the qualitative ones consist in the reduction of muscle strength and in physical performance. Among qualitative changes of uremic muscle should include alterations of muscle mitochondrial morphology, their protein pathways, and decreased mitochondrial respiratory function [9,10]. Moreover, the uremic condition predisposes to a capillary rarefaction altering physiological muscle function [11]. It has also been observed that aging, in addition to the loss of muscle strength and mass, is associated with increase in fat mass, especially localized in the abdominal area [12]. Many studies conducted on changes in musculature, during lifespan, are transversal. The data obtained overall indicate an estimated reduction in MM by about 1–2% *per* year after 50 years and muscle strength declines by 1.5% between ages 50–60, with a tendency to a further reduction of up to 3% *per* year, thus achieving a total loss of MM and muscle strength of about 40% from 30 to 70 years of age [8]. Longitudinal studies in geriatric populations confirmed the results of these cross-sectional studies, as discussed below. A study by Delmonico et al. [12], conducted on 1678 subjects (heterogeneous for age, sex and ethnicity), showed that from 70 years of age there is an annual reduction of the muscular area of 4.9 ± 7.4% in men and 3.2 ± 7.9% in women. Similar results were obtained by Cameron et al. [13] in a population of seventy, in which lean mass was measured with magnetic resonance imaging (MRI) and dual-energy X-ray absorptiometry (DXA). The loss of MM was approximately 5% in five years of follow-up regardless of basal MM values. The phenomenon of aging is characterized by a primary reduction in muscle strength and subsequently occurs a reduction in MM. This could be due to the progressive infiltration of adipose tissue into the muscle [14] and the development of muscle fibrosis [15], processes that reduce muscle performance but do not result in a reduction of muscle volume.

Since 2010 sarcopenia has gained more interest in the scientific community, thanks to the publication of the first consensus on the subject published by the European Working Group for Sarcopenia for Older People (EWGSOP) [16]. The criteria to be considered are three: 1—reduced MM, 2—reduced muscle strength, 3—reduced physical performance. According to the EWGSOP, the presence of criterion 1, associated with criterion 2 or 3, is required for the diagnosis of sarcopenia. Over the years, consensus has developed by different societies that agree in defining sarcopenia as a syndrome characterized by the progressive and generalized loss of MM and strength, associated with the high risk of adverse events such as physical disability, reduced Qol and death. The first consensus was revised in 2019 and the main parameter considered for the diagnosis of sarcopenia is no longer MM but muscle strength [16,17,18,19,20]. The EWGSOP1 [16] divided sarcopenia into primary, when this condition is related to age in the absence of other obvious causes, and secondary, when it is determined by other pathological conditions and not necessarily by advanced age. Secondary sarcopenia can occur in conditions of reduced physical activity (lodging, sedentary life, zero-gravity conditions), in diseases (CKD, inflammatory diseases, endocrine and malignant diseases) and under conditions of reduced intake of nutritional factors (reduced intake of nutrients, malabsorptive conditions, gastrointestinal diseases, use of anorexic drugs) [21,22]. The main difference between primary and secondary sarcopenia is that in the former the loss of MM occurs consistently and generally continues from the fourth to fifth decade, while in secondary sarcopenia the loss of MM is not only related to advancing age, but also to the development of pathological processes of protein degradation that are more aggressive than those that occur physiologically with aging [23]. The EWGSOP2 defined the concept of acute and chronic sarcopenia, considering the first as an acute condition present for less than six months, while the second is considered a chronic condition present for more than six months. Acute sarcopenia is often linked to an acute disease; on the contrary chronic sarcopenia is correlated with a chronic and progressive disease that induces an increased risk of mortality [20]. Experimental data showed that muscle tissue should be investigated through the execution of biopsies which permits the detection of distinct variations between primary and secondary sarcopenia [24]. In fact, primary sarcopenia is generally characterized by atrophy of both type I and type II fibers, while secondary sarcopenia, especially that associated with CKD, causes a specific reduction of type II fibers. In any case, these data need to be confirmed by further research. Secondary sarcopenia is often associated with two other well-known pathological conditions: protein-energy wasting (PEW) (present in between 18% and 75% of chronic hemodialysis (HD) patients [25,26] and cachexia. PEW is a condition of malnutrition characterized by a reduced and inadequate protein and energy intake. It is a multifactorial condition whose diagnosis is complex and involves the integration of laboratory and anthropometric parameters. The International Society of Renal Nutrition and Metabolism (ISRNM) has defined a set of criteria necessary for the diagnosis of PEW [27]. These criteria are body mass index (BMI) < 23 kg/m^2^; unintentional body weight loss of about 5% in 3 months or 10% in 6 months; reduction of arm circumference > 10% compared to the 50° percentile of the reference population; MM reduction of about 5% in 3 months or 10% in 6 months; albuminemia < 3.8 g/dL; total cholesterol < 2.59 mmol/L; unintentional protein intake < 0.8 g/kg *per* day in HD patients; unintentional energy intake < 25 kcal/kg *per* day. Cachexia is a condition of metabolic imbalance characterized by the loss of MM with or without loss of adipose tissue [28,29]. The Society of Sarcopenia, Cachexia and Wasting Disorders (SCWD) [28] has proposed a series of criteria necessary for the definition and diagnosis of cachexia: albuminemia < 3.2. g/dL; hemoglobin < 12 g/dL; increment of inflammatory markers or C-reactive Protein (CRP) > 5 mg/L, interleukin (IL)-6 > 0.4 pg/mL; BMI < 20 kg/m^2^; unintentional loss of 5% of body weight in 12 months; appendicular skeletal muscle index, measured with DXA, <7.25 kg/m^2^ in men and <5.45 kg/m^2^ in women; reduced arm circumference; “fatigue” understood as physical and/or mental fatigue resulting from the effort or inability to continue an exercise with the same intensity, resulting in a worsening of performance; reduced appetite; unintentional reduction of energy intake < 20 kcal/kg *per* day; 70% reduction in usual daily energy intake [28].

Sarcopenia should be considered not only as a geriatric disease, but a multidisciplinary condition. Since the first consensus on sarcopenia, published in 2010 by the EWGSOP [16], numerous studies have been performed to investigate the possible correlations of sarcopenia with CKD, especially with ESRD [30,31,32]. It has been shown that secondary uremic sarcopenia is determined by a more severe protein degradation process with respect to primary sarcopenia [33]. In primary sarcopenia, it is essential to restore a proper motor activity and an adequate Qol, thereby reducing the mortality rate. In secondary sarcopenia, where “muscle wasting” and PEW are prominent, the main goal is to reverse the process that causes sarcopenia or to restore optimal nutritional status. The latter allows patients to respond more effectively to therapeutic treatment. Further therapeutic objectives are to restore appropriate mobility and Qol and to reduce mortality and hospitalization rates. In 2019, EWGSOP2 [20] continues to define sarcopenia as a concomitant presence of altered quantity and quality of MM but, differently from the previous consensus, EWGSOP2 proposed the use of reduced muscle strength as a key criterion to identify “probable-sarcopenia”. The diagnosis of sarcopenia is confirmed by the presence of low muscle strength and low muscle quantity or quality. The stage of severe sarcopenia is reached when the patient presents a reduction in MM (quality and quantity), muscle strength and physical performance simultaneously. According to EWGSOP2, therefore, reduced MM is no longer the key element in the diagnosis of sarcopenia, but reduced muscle strength. This change is justified by the fact that reduced muscle strength is better than reduced MM in predicting adverse outcomes in sarcopenic patients [34]. Leong et al. [34], in fact, measured the prehensile force of the hand of 142,861 subjects whose age was between 35–70 years. After a follow-up of 4 years, the authors observed that the degree of prehensile strength of the hand was inversely related to all causes of mortality, cardiovascular (CV) and otherwise, and to the onset of acute myocadiac infarction and stroke. This study confirmed that the simple dynamometer measurement of the prehensile force of the hand would provide information about the patient’s prognosis, since it would relate to all causes of mortality. Thanks to the simple use of the portable manual dynamometer, muscle strength is therefore easily detectable, not only in hospital facilities, but also in other care centers. Another method for assessing muscle strength characterized by easy execution is the chair stand test as defined by the EWGSOP2. The chair stand test evaluates the muscle strength of the quadriceps group and its proper execution requires both strength and endurance from the patient. This test is adjuvant in the early diagnosis of sarcopenia [20]. 

In this review we discuss the uremic sarcopenia condition and the possible therapeutic strategies currently available, with particular interest in nutritional therapy. The research was conducted on Medline (Pubmed) including studies published until April 2020. The terms used for the research were: “uremic sarcopenia”, “nutritional therapy”, “chronic kidney-disease”, “CKD”, “dialysis” and “hemodialysis”.

## 2. Sarcopenia in Chronic Kidney Disease

**Figure 1:**
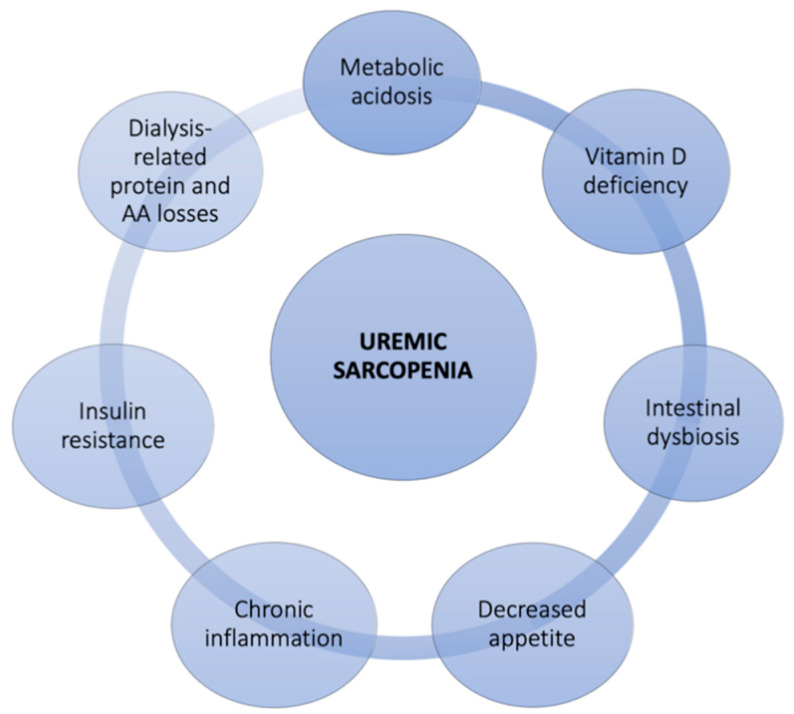
Possible causes of uremic sarcopenia. *AA*, *amino acids*.

Loss of MM is common in CKD, especially in ESRD patients in hemodialytic treatment [2,3,4,31]. In this population, the consequences of MM loss are not only related to reduced physical ability, as happens in the elderly population. Many studies in fact have associated the loss of MM in CKD to the reduction of Qol, to the manifestation of PEW, to fractures, to CV complications, to loss of graft and to postoperative complications in kidney transplant [35,36]. These conditions together lead to an increased risk of hospitalization and mortality in the nephropathic patient with sarcopenia [25,37,38]. For this reason, sarcopenia can be considered a negative prognostic factor in CKD patients and only an adequate knowledge of this pathology and the mechanisms involved in its genesis can allow a valid and effective treatment of the same [39]. The possible etiological factors involved in MM loss in CKD patients are various, as reported in Figure 1.

**Figure 2:**
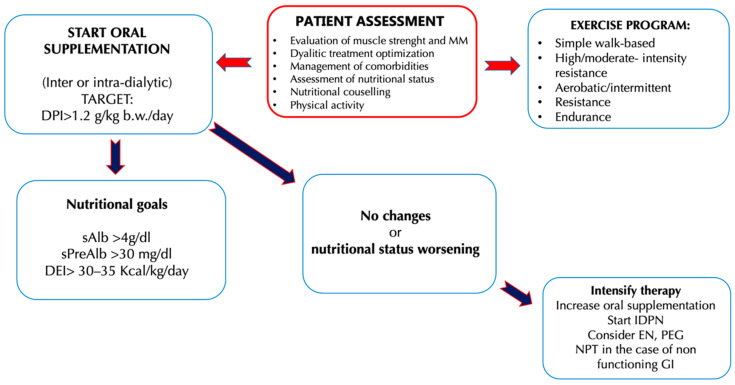
Clinical management of hemodialysis (HD) patients to counteract the onset and the progression of uremic sarcopenia [91]. Abbreviations: b.w., Body weight; DEI, Diet energy intake; DPI, Diet protein intake; EN, Enteral nutritional; GI, Gastrointestinal; IDPN, Intra-parenteral nutrition dialysis; MIS, Malnutrition inflammation score; MM, Muscle mass; PEG, Percutaneous endoscopic gastrectomy; SGA, Subject global assessment; TPN, Total parenteral nutrition.

Major causes include metabolic acidosis, dialysis therapy, and typical low-grade chronic inflammatory status [40,41]. These elements together result in an increase in protein degradation and in a reduction in protein synthesis, producing a negative nitrogen balance (Figure 2) [42,43]. CKD, in addition to the development of metabolic acidosis, is associated with the onset of insulin resistance (IR) and vitamin D deficiency, and these factors can also act as promoters of protein catabolism and of reduced protein synthesis [44,45,46,47]. It has been observed that metabolic acidosis acts as a powerful stimulator of protein catabolism thanks to the solicitation of two systems responsible for intracellular protein degradation: caspase 3 and the ubiquitin proteasome system (UPS) [48]. In particular, it has been shown that caspase 3 is responsible for the cleavage of actomyosin of myofibrils generating actin fragments from 14 kDa. Workeneh et al. [49] performed muscle biopsy on 28 patients in chronic hemodialysis treatment. The authors noted that patients with malnourished and reduced physical activity had high levels of actin from 14 kDa to muscle biopsy.

Metabolic acidosis seems to be related to increased IR and to the enhancement of insulin-like growth factor-1 (IGF1) [50]. IGF1 is an important regulator of muscle turnover. In fact, through the suppression of the PI3K pathway, it determines the increased activation of the ubiquitin ligases enzyme (E3s), that activates the UPS protein degradation system [51,52]. CKD, particularly in the presence of metabolic acidosis, is associated with resistance to the action of growth hormone (GH), the anabolic hormone responsible for the turnover of skeletal muscle cells. This condition, present in CKD patients, alters the balance between anabolism and catabolism of the MM [53].

Vitamin D deficiency in experimental studies has been shown to be involved in reduced insulin secretion by beta pancreatic cells, participating in the IR process [54,55]. Vitamin D deficiency also appears to decrease protein synthesis, reducing muscle expression of vitamin D receptors and changing intracellular calcium flow, leading to an altered functionality of muscle cells [46]. The renal dysfunction is associated with many hormonal alterations including the increase of cortisol and the reduction of testosterone [56,57]. Cortisol in vitro studies has been shown to possess the ability to activate the UPS system and to suppress the PI3K pathway, leading to an increase in protein degradation at the muscular level, thus participating in the development of sarcopenia [58]. 

The action of testosterone is related to skeletal muscle tropism. In CKD patients and, in particular, in HD patients, there is a reduction of this sexual hormone with a reduction in MM and strength. In fact, the reduction of testosterone induces an increased expression of myostatin (protein that inhibits muscle growth), and an alteration of IGF-1 mediated signaling [59].

HD treatment is a determining factor in the altered degradation/protein synthesis ratio. During the HD session, a substantial loss of proteins and amino acids is observed, with a reduction in the availability of nutrients for the synthesis of muscle proteins. It has been calculated that, during a single dialytic session, up to 5–8 g of free amino acids can be lost [60]. It has also been shown that, during and at the end of dialysis treatment, there is increased protein degradation [61]. In addition to the loss of protein during dialysis session, there is also a tendency of HD patients to a reduced spontaneously energy and protein intake, above all in the day of HD treatment [62], determining a state of energy and protein imbalance. In fact, CKD patients presented a reduced sense of appetite related to multiple factors such as sedentary life, high level of uremic toxins [63,64], the chronic inflammatory state, and hormonal disorders. Among these can be mentioned the reduction of ghrelin and various neuropeptides (neuropeptide Y) and the increase in leptin levels [65]. The chronic inflammatory condition present in HD patients, due to increased protein catabolism, is related to various factors, first of all, the pro-inflammatory response of the organism induced by the dialyzer, despite the use of membranes with high biocompatibility. Tumor necrosis factor (TNF)-α appears to be the cytokine most responsible for activating protein catabolism processes and for increasing the anabolic processes, as it results in increased action of caspase-3 and suppression of insulin action [66].

Chronic HD patients usually show high levels of other pro-inflammatory cytokines such as Interleukin (IL)-6 often associated with an increase in serum CRP. IL-6 in association with TNF-α reduces the suppression of cytokine signaling (SOCS)-3 proteins by determining the intracellular activation of UPS and caspase-3 [67]. 

IL-6 also accelerates muscle degradation by stimulating the expression of the signal transducer and transcriptional activator-3 (Stat3) responsible for the altered expression of myostatin. IL-6 would result in muscle loss [68].

In recent years, the development of a systemic and chronic inflammatory state has also been attributed to gut microbiota alterations [69]. It has been observed that in CKD patients gut dysbiosis occurs due to the production of toxins harmful to the integrity and functionality of cells of the enteric tract. Among these, p-cresol sulfate and indoxyl sulfate induce an inflammatory cascade, which in turn induce a low-grade systemic inflammation. The alteration of the gut microbiota, therefore, participates in the development of the chronic inflammatory state typical of CKD. Dysbiosis is produced mainly as a result of exposure of the intestinal mucosa to a uremic environment and reduced fiber intake due to dietary restrictions typical of HD patients (reduction of potassium-rich foods but also of fibers such as fruit, vegetables and whole grains) [70]. CKD patients often reduce physical activity that causes not only increased CV mortality but also a progressive reduction in MM. This MM reduction is described as “atrophy from disuse” and is related to an increased risk of developing sarcopenia. For this reason, physical activity is one of the main therapeutic strategies for sarcopenia, and its intensity in duration and frequency depends on the severity of the CKD and the clinical conditions of the patient [71]. In HD patients, an activation of caspase 3 that induces an increased muscle proteolysis has been observed. This factor in association with intracellular apoptosis can contributes to MM loss in CKD patients [49,72]. CKD patients are often elderly, and age is a prominent factor in the development of sarcopenia. In CKD patients, therefore, the risk of developing sarcopenia is greater when compared to individuals of the same age in the general population. CKD was also defined as a “premature aging” model [73,74] and, for this reason, nephropathic patients often experience a reduction in MM at an earlier age than the general population, thereby increasing the risk of disability and mortality.

The development of sarcopenia in CKD patients leads to reduced Qol and autonomy, but especially an increased risk of complications and mortality. It is clear that the use of appropriate methods for its diagnosis and the setting up of an effective therapy to restore an increase in MM are necessary to prevent and treat this condition, and to improve its prognosis *quoad vitam*.

### 2.1. The Diagnosis of Sarcopenia

The methods available for the evaluation of the three main parameters useful for the diagnosis of sarcopenia (muscle strength, MM and physical performance) are several and include simple and cheap or more accurate but expensive methods. The consensus of the EWGSOP describes the different diagnostic methods available for the evaluation of the three principal parameters. The first EWGSOP1 consensus [16] focused on the condition of sarcopenia and quantitative alterations of MM. The second, revised in 2019, (EWGSOP2) [20], updated the criteria of sarcopenia diagnosis based on the reduction in muscle strength. With regard to the evaluation of MM, several diagnostic methods can be used such as computed tomography (CT), MRI, DXA, or bioimpedance analysis (BIA). Currently, CT and MRI are considered the gold standard methods. Both methods allow not only the evaluation of quantity but also of muscle quality. Their limitation is that not all clinical centers have such methods available and their use exposes the patient to radiation. A valid alternative at low cost could be DXA, that allows the evaluation of fat mass, lean mass and bone mineral density [75]. Unfortunately, even this method is not present in all centers and in CKD patients does not allow a distinction between MM and extracellular fluid, thus limiting its use in this population [76]. BIA is a tool with simple use and low cost for the estimation of lean and fat mass. It is therefore a valid alternative to DXA for the evaluation of body composition, although in CKD patients the inflammatory state and the excess of extracellular fluid could alter the results [77]. 

For the study of muscle strength, “functional” tests are used: grip force, the flexion and extension of the muscles of the thigh, and forced expiratory flow. The test most commonly used in clinical practice is that of handgrip strength (HGS). HGS is related to lower limb muscle strength and is considered a predictor of reduced overall MM [78]. The methods to be used in clinical practice, recommended by the EWGSOP for the evaluation of physical performance, are the usual gait speed, the timed get-up-and-go test and the short physical performance battery (SPPB) [16,79]. The gait speed test evaluates the time taken to walk 4 m, while the timed get-up-and-go test measures the time taken to get out of a chair, walk three meters, turn around and return to the chair to sit again. The SPPB assesses the functionality of the lower limbs and includes a test battery divided into three different sections. The first section allows the evaluation of the equilibrium through three tests: the maintenance of the joined foot position for 10 s, the maintenance of the semi-tandem position for 10 s (big toe of the foot placed laterally to the heel of the counter-lateral foot) and the maintenance of the tandem position for 10 s (toe of the foot placed behind the heel of the foot against the side). The second section includes the usual gait speed test. The third section investigates the ability to perform, five consecutive times, the sit-to-stand test, or the ability to stand up and sit down from a chair without the help of the upper limbs.

Pereira et al. [30] conducted one of the few studies aimed at defining criteria for the diagnosis of sarcopenia, in CKD patients in particular, in conservative therapy. According to the authors, the diagnosis of sarcopenia in nephropathic patients is defined by the presence of: a reduced muscular function evaluated by HGS (HGS 30% percentile of the reference population adjusted for sex and age); reduced MM assessed by three different methods, namely arm circumference (mid arm muscle Circumference—MAMC), 90% of the reference value, loss of MM estimated by Subject Global Assessment (SGA), and finally a reduced skeletal MM index (<10.76 kg/m^2^ in men; <6.76 kg/m^2^ in women) estimated by BIA.

### 2.2. Possible Therapeutic Strategies for Uremic Sarcopenia

There are many current treatments available to implement MM and improve physical performance in patients with uremic sarcopenia. In this review, the role of nutritional therapy will be deepened, whose role has been object of study and experimentation, especially in recent years, providing decidedly promising results in the treatment of this pathological condition. Finally, the other main therapeutic options available according to the scientific literature will be dealt with, with a final reference to possible future therapeutic strategies.

## 3. Nutritional Therapy

Dietary interventions necessary to preserve MM and provide an adequate energy and protein load are essential for the health of HD patients and their nutritional status, especially when the processes of MM reduction are already established [80]. For CKD (stages III–V) patients, a protein intake of 0.6–0.8 g/kg body weight (b.w.)/day and an energy intake of 30–35 kcal/kg b.w./day is recommended. Despite the risk of a negative nitrogen balance, a low protein diet (LPD) is indicated for CKD, because it is related to a better metabolic status and a better control of the signs and symptoms of CKD, if compared to a diet with high protein intake [81,82]. The risk of protein restriction consists in the possibility of establishing a state of malnutrition, complicated by the reduction of MM, and therefore by sarcopenia. For this reason, it has been proposed to combine the LPD with the use of energy supplements with controlled fats and carbohydrates content [83,84]. In this regard, a study by Wu et al. [84] was conducted on 109 CKD patients (stage III-IV), divided into a control group (54 patients) and an experimental group (55 patients). For a duration of 24 weeks, all participants were prescribed a diet with a controlled protein intake (CPI), comprising between 0.6–0.8 g/kg b.w./day and an energy intake of 30–35 kcal/kg b.w./day, associated with dietary counseling. Only the experimental group was required also to consume an energy supplement of 200 kcal/day (40 g of maltodextrin together with 5 g of oil creamer). The authors noted that the patients in the experimental group, thanks to the energy supplementation, had a greater adherence to the prescribed nutritional therapy and, therefore, better values for the renal function indices. Urinary protein excretion values were significantly lower respect to the control group, as well as creatinine and azotemia values, while glomerular filtration rate (GFR) values were higher. This study suggested that CKD patients in conservative therapy can successfully benefit from a diet with a CPI. At the same time, a controlled energy supplementation would ameliorate, on the one hand, the adherence to the same diet with CPI, and on the other would improve the nutritional status of the CKD patient.

The LPD, with or without energy supplementation, can be used successfully especially in younger CKD patients. In fact, the protective effects of the LPD on the progression of CKD were less evident in the geriatric population. An example of this is the study of Levine et al. [85] that examined the epidemiological data from NHANES III—National Health and Nutrition Examination Survey [86]. NHANES III is a cross-sectional study conducted to assess the health and nutritional status of a representative sample of the US population for ethnicity, education and comorbidity (6381 subjects aged over 50 years). From the observation of such data, the authors have evidenced that the mortality rate among the individuals with LPD was different, depending on the age of the subjects analyzed. In particular, individuals between the 50–65 years who followed a high-protein diet (HPD) had a significant increase in mortality for all causes up to a value of 74% compared to individuals of the same age but who followed a LPD. These results were opposed if the subject of the study were individuals over 66 years of age. This group, on the contrary, presented a 60% reduction in mortality rate for all causes if it consumed a LPD, while the reduction was only 28% if it consumed a diet rich in protein. This study shows that in the geriatric population higher protein intake is related to a better prognosis due to a reduced risk of mortality. The reduced protein intake in CKD patients must always take into account the possible risks of malnutrition associated with this dietary restriction, therefore the LPD must be prescribed by a multidisciplinary team that includes a nephrologist, a nutritionist expert in renal diseases and a psychologist, the latter necessary to allow the acceptance of chronic pathology and increase adherence to the prescribed dietary-nutritional treatment. It has been observed that malnutrition in CKD patients is associated with a faster progression of kidney disease, and thus a worsening of the prognosis with an increase in the mortality rate [87]. The recommendations for protein intake in ESRD patients reflect the need to ensure an adequate nutritional intake to prevent the development of comorbidities such as sarcopenia, PEW and cachexia. The recommendations of the current guidelines and consensus published so far is to take at least 1.1–1.2 g/kg b.w./day of protein for ESRD patients under HD treatment [81,88,89,90]. In HD subjects, a higher protein intake is therefore recommended compared to the one advised to the general population (0.8/kg b.w./day), especially considering the loss of amino acids and proteins that occurs during HD treatment. Moreover, in HD patients an intake of 35 kcal/kg b.w./day is recommended, taking into consideration also the age, the physical activity carried out by the patient and the hypercatabolic state induced by dialysis [81,88,89,90].

In ESRD, uremic intoxication and the need to slow the progression of nephropathy are no longer the focal point of nutritional therapy, as they are treated by adequate HD therapy. The prevention and management of MM reduction thus becomes the focal point of nutritional therapy that is focused on maintaining an equal or positive nitrogen balance, so as to ensure better outcomes in patients in HD treatment. Sabatino et al. [91] have proposed an interesting algorithm that indicates the intervention times on the nutritional status, the types of intervention, and the protein-energy intake targets to be achieved in chronic HD patients. The need to further implement the protein intake in HD patients is due to the presence in this category of an increased protein catabolism and reduced synthesis that induces the development of pathological conditions such as sarcopenia. In addition, as emerged from a study of Cupisti et al. [92], often up to 50% of HD patients assume less than 1 g/kg b.w./day protein and experience a reduced energy intake, aggravating their negative nitrogen balance condition.

Several studies have been carried out to investigate the different strategies that ensure an adequate protein-energy intake in HD patients. Numerous studies have shown that, in nephropathic patients, HPD causes an accumulation of toxic compounds (derived from protein metabolism), while a LPD offers better outcomes in these patients [93]. In particular, in CKD it is necessary to distinguish two categories of patient: the first (i) is represented by patients undergoing conservative therapy and the second (ii) is represented by patients in renal replacement therapy. In the first category, an HPD, defined as a diet with an intake greater than 1.2 g prot/kg/b.w. *per* day, is able to alter renal hemodynamics, inducing the condition of hyperfiltration [94]. Renal hyperfiltration consists in the increase in renal blood flow inducing an enhancement in intra-glomerular pressure that causes a GFR increase and the elimination of protein-derived waste nitrogen products. This cause an increase of renal parenchyma volume and body weight [94]. An HPD is also associated with an increase in urinary excretion of albumin or protein, which induces consequences both at the level of the kidney and at the level of other organs [95]. The presence and degree of albuminuria, according to current guidelines, are related to the severity of CKD and to CV mortality risk. Proteinuria induces apoptosis of renal tubular cells and alters the regeneration of podocyte cells, inducing tubular atrophy that favors the progression of kidney damage [96]. A further effect induced by an HPD is that related to metabolic acidosis. Protein metabolism is able to generate acids, derived from the metabolism of sulfide amino acids, and this phenomenon induces a decrease in bicarbonatemia worsening the picture of metabolic acidosis typical of CKD. In the literature, the phenomenon that correlates acid retention with the decline of kidney function has been described, inducing the chronic condition of metabolic acidosis [97]. This phenomenon alters protein metabolism, increases muscle catabolism and loss of muscle mass, worsens residual kidney function and, simultaneously, uremic symptoms [98]. Gaggl et al. [99] demonstrated a positive correlation between metabolic acidosis and the assumption of sodium bicarbonate in IV-stage CKD patients. In fact, they demonstrated that sodium bicarbonate oral supplementation was able to slow the decline of kidney function and improve nutritional status, with two years follow-up. HPD is also related to high phosphorus intake, mainly content in animal origin proteins and in food additives [100]. Its high blood levels help to induce the alteration of calcium-phosphorus metabolism, a very frequent condition in CKD patients [92]. The increase in phosphoremia is directly related to high levels of parathyroid hormone and fibroblast growth factor (FGF)-23 [101], and induces an alteration of the vasal walls. The latter increases the risk of CV disease [102]. Therefore, in this patient population, the administration of HPD would not be a valid therapeutic tool since it would lead to a faster decline in kidney function. In the second category of patients, it is necessary to consider the impact of dialysis treatment on protein metabolism in order to determine the correct daily protein intake *per* kg of b.w. Current guidelines [103] for HD patients recommend a high protein intake, as previously mentioned, because dialytic procedure stimulates protein catabolism with high the risk of MM loss. Therefore, in HD patients a high protein intake is necessary in order to avoid the establishment of PEW syndrome, induced by the loss of amino acids and proteins during the dialysis treatment. In fact, it is possible to observe in ESRD patients the phenomenon of “obesity paradox”, highlighted as a higher BMI reduces the risk of mortality [104]. 

The HPD seems to be inversely related to the onset of the fragility state [105]. Fragility is defined as having at least three of the following five symptoms: weight loss, low physical activity, asthenia, slowing down, and fatigue. This condition is characterized by an increased susceptibility to adverse health events, leading to an enhancement incidence of hospitalization, falls and consequent fractures, disability, request for nursing assistance, and death [106,107,108]. Therefore, a diet associated with physical exercise plays a key role in the prevention and treatment of frailty [109]. All studies agree on the importance of early and regular dietary counselling to ensure a better success of nutritional therapy, especially in terms of adherence to the same [91]. Nutritional counseling must provide correct dietary nutritional information, evaluate past eating habits and identify the presence of any deficit in protein or energy intake. In addition, for patients with an energy intake less than 30 kcal/kg/day or protein intake less than 1 g/kg/day, it is necessary to provide information and tools to increase intake. Correct indication must be provided to avoid foods with high content of phosphorus, potassium and sodium and to try to avoid unnecessary fasting periods, as may occur after dialysis or in periods of acuity or hospitalization. It is therefore appropriate to reiterate the concept that a multidisciplinary team assesses the risk of uremic sarcopenia and identifies the best protein intake for each patient [92]. The therapeutic possibilities identified so far to provide a proper protein–energy intake in HD patients are manifold and include the use of oral nutritional supplements (ONS), intra-parenteral nutrition dialysis (IDPN), enteral nutrition (EN) or total parenteral nutrition (TPN). Other useful tools proposed are specific nutritional products such as fibers and ω-3 (Table 1) [91].

### 3.1. Oral Nutritional Supplements

As mentioned in Figure 2, ONS are the first step to be taken when a reduction of the protein-energy intake by the patient is highlighted. Generally, a standard ONS ensures the achievement of nutritional targets, as it provides up to 10 kcal/kg b.w./day and 0.3–0.4 g/kg b.w./day of protein, if consumed twice a day. It is essential that the ONS provides a high energy intake (1.8–2 kcal/mL), so as to reduce the risk of overload of liquids, a dangerous event for HD patients. In addition, the ONS are generally formulated to present a pleasant taste and good palatability for the patient in order to ensure assumption [91].

HD patients are often elderly and have issues related to appetite reduction or swallowing, therefore the use of ONS with high protein and energy content in the form of small-volume snacks has clear advantages in terms of spontaneous assumption of the nutritional supplement [120,121].

Consumption of ONS may be intra-dialytic or inter-dialytic. Intra-dialytic administration is usually preferred because it is associated with greater compliance of the patient, not interfering with the intake of the conventional meal at home [91].The main function of ONS is to provide a protein load to counteract the protein catabolism associated with HD treatment [60]. According to various studies, ONS have demonstrated their effectiveness not only in the improvement of nutritional status but also in inflammatory indices, in physical performance and in prognosis and lower risk of mortality [110,111,112,113,114,122]. 

Lacson et al. [111] conducted an observational study lasting one year on a large sample of HD patients, characterized by an albuminemia of 3.5 g/dL. These patients were divided into a control group and an experimental group, to which an ONS was administered three times a week during HD treatment. Each group included 5227 patients, respectively. The authors highlighted that those who assumed ONS treatment had a protein intake increase, an enhancement of albuminemia (an important biomarker of nutritional status), but primarily a higher survival rate than the control group. 

Similar data emerged from the Sezer et al. study [112], conducted on 62 chronic HD patients divided into a control group and an experimental group to which an intra-dialytic ONS was administered. The authors, in a follow-up period of six months, showed in the experimental group a progressive increase in albuminemia values and in body weight that instead was reduced in the control group. An interesting aspect emerged from this study is that the patients in the experimental group, thanks to the achievement of a better nutritional status, presented a reduced need for therapy with recombinant human erythropoietin (EPO). The ONS thus demonstrate to have multiple beneficial effects, specifically including the ability to allow a reduction of EPO therapy, a fundamental tool for the anemia treatment in the CKD patient.

Caglar et al. [110] conducted a study of 85 HD patients who were given an ONS during the HD session for a period of six months. The authors observed a significant and progressive increase of albumin and prealbumin and a SGA improvement in the group that assumed ONS. The SGA [123] is a diagnostic tool that through a series of specific questions about the clinical conditions and the patient’s history allows a detailed assessment of nutritional status. Based on the results obtained, SGA divides patients into three categories: A—well-fed, B—moderately malnourished, C—severely malnourished. In conclusion, the study of Caglar et al. [110] has shown that the use of ONS is associated with an improvement in nutritional status, detected through laboratory parameters but also through an evaluation of the clinical state, detected with SGA. HD patients are hospitalized on average twice a year, and up to 35% of these are re-hospitalized within 30 days of the first discharge [122]. Some studies have demonstrated that the use of ONS is able to reduce the hospitalization rate in HD patients. In this regard, Benner et al. [113] conducted a study on a large sample of HD patients with hypoalbuminemia (albuminemia < 3.5 g/dL) divided into an experimental group (n = 3374) who assumed an ONS vs. control group (n = 3374). The study highlighted that the use of ONS, during HD treatment, induced a mortality reduction of up to 69% and a significant increase in protein intake. Another interesting fact emerged from the study, in that ONS caused an increase in body weight but especially a reduction of up to 33% of the number of dialysis sessions considered “lost” or not performed. Hospitalization results in a failure to conduct the dialysis session at the reference center. According to the authors, a better nutritional status would therefore be related to an improvement of outcomes and therefore a reduced risk of hospitalization.

Leonberg-Yoo et al. [114] conducted a study of 5479 patients in chronic HD who were hospitalized (for any reason) during the 12 months before the start of the study and with hypoalbuminemia at the time of discharge (albuminemia < 3.5 g/dL). The experimental group receiving ONS consisted of 1420 patients, and 4059 patients received no ONS (control group). The study showed that the control group presented a risk of re-hospitalization within 30 days of the first discharge, significantly higher than the experimental group. The association between the use of ONS and the reduction of the hospitalization rate is an important factor for the HD patient. The latter, in fact, is often elderly and suffering from multiple comorbidities, and is therefore considered a “fragile” individual. The use of ONS is a possible tool to reduce the hospitalization rate, decreasing intra-hospital complications and consequently the risk of mortality in HD patients.

Some studies have demonstrated that ONS assumption not only induced a nutritional benefit but also a better prognosis with a reduced risk of hospitalization and mortality [113,124].

### 3.2. Amino Acids Supplementation

A further nutritional strategy useful in countering the onset of, and improving, uremic sarcopenia is the administration of keto-analogues (KAs). The latter are essential amino acid nitrogen-free analogs [125]. According to previous studies, a LPD in combination with essential amino acids and KAs is useful in maintaining good nutritional status in patients with advanced nephropathy [126]. In fact, in order to prevent inadequate intake of amino acids, it is mandatory to supply the optimal amount of these nutrients. The mechanisms responsible for muscle alteration are represented by an increased OS and by mitochondrial dysfunction associated with an up-regulation of p66SHC and Forkhead box O3A (FoxO3A). A study in CKD mouse models conducted by Wang et al. showed that KAs supplementation is able to improve muscle atrophy and reduce OS and mitochondrial damage [127]. 

A subsequent study conducted by Zhang et al. [128] in CKD mouse models evaluated the effects of KAs supplementation on autophagy and inflammation of skeletal muscle. The authors concluded that the LPD diet supplemented with KAs improves autophagy but does not appear to have any effect on the inflammatory state. 

A study conducted on CKD rats evaluated the effect of KAs on the mechanisms of prevention of muscle atrophy [129]. The authors divided the study population into 3 subgroups fed for 24 weeks with the following dietary treatments: 1—normal-protein diet-group, 2—LPD group and 3—LPD + KAs group. The results obtained showed that KAs improve protein synthesis and inhibit ubiquitin-proteasome mechanisms. They also reduce DNA fragmentation in muscle cells, confirming their action as a supportive therapeutic strategy to counteract muscle atrophy.

A further protocol of KAs supplementation is that obtained by combining amino acids with the KAs [130]. In a study conducted by Barsotti et al., the authors demonstrated that a LPD supplemented with KAs and amino acids allowed a slowing of the progression of renal disease and reduced CV risk. This finding was explained by the fact that the supplemented LPD carried a low amount of phosphorus which in turn induced a negative balance so as to determine a decrease in serum phosphorus [131]. 

A subsequent study by Zemchenkov et al. of 96 CKD patients with stage 3b-5 confirmed that an LPD supplemented with essential amino acids and KAs induced a slowdown in the progression of CKD, particularly in female elderly patients and in those with low levels of plasma phosphorus [132].

### 3.3. Intra-Dialytic Parenteral Nutrition

IDPN consists of the administration of an amino acids, glucose and lipids mixture through the venous line of the dialysis circuit during each session of HD. The IDPN presents a shortening of the administration time depending on the frequency and duration of the dialysis session. A safe and effective IDPN for a patient with an average weight of 75 kg should consist of no more than 1 L of infusion fluid (to avoid water overload) and a maximum of 1000 kcal and 50 g of amino acids [133]. The choice to administer IDPN depends on the difference between the value of the protein-energy intake to be reached and the protein intake energy taken by the patient. The maximum nutrient intake that can be obtained with IDPN is about 3000 kcal and 150 g of amino acids, that is 5 kcal/kg b.w./day and 0.25 g/kg b.w./day of amino acids in a patient of about 70 kg. Since IDPN can guarantee up to a maximum of 25% of the ideal nutritional intake, it may only be administered if the patient has a spontaneous protein intake of 0.8–0.9 g/kg b.w./day and an energy intake of 20 kcal/kg/day [89,133].

IDPN cannot be considered as a long-term treatment. After the achievement of the predetermined targets that usually takes place within 3–6 months from the start of the IDPN, its suspension and continuation of nutritional treatment with the ONS is required [89,133]. Intra-dialytic ONS could therefore be useful to ensure an adequate protein and energy intake in HD patients, compensating for the loss of amino acids that occurs during HD treatment and counteracting the catabolic effect of HD itself [60,61].

The criteria that allow stopping of therapy with IDPN and implementation of the switch to ONS are: albuminemia values stable > 3.8 g/dL for more than three months; improvement of the SGA score up to stage A (well nourished) or B (moderation malnourished); positive clinical examination for effective nutritional improvement; increased protein intake > 1 g/kg b.w./day and energy > 30 kcal/kg b.w./day [133].

The studies conducted so far concerning the effectiveness of IDPN in HD patients are encouraging. Marsen et al. [115] conducted a prospective multicenter randomized study of 107 malnourished patients in chronic HD (SGA stage B—moderate malnutrition or stage C—severe malnutrition) divided into a control group and an experimental group, to which IDPN was administered three times a week for 16 weeks. The observation period continued for 12 weeks after the end of nutritional therapy. The authors noted that the administration of IDPN was associated with a significant increase in pre-albumin, which was stable in the weeks following the discontinuation of the IDPN. This was even more evident in patients with moderate malnutrition (SGA stage B). IDPN would therefore be responsible for the improvement of pre-albumin values, whose reduction is a known negative prognostic marker for CKD patients [134]. The study highlights the importance of early intervention to prevent or treat malnutrition. Despite that IDPN is a more invasive nutritional intervention than ONS, it still has less effectiveness in conditions of serious malnutrition that must be carefully avoided by using early nutritional counselling. The improvement of pre-albumin thanks to IDPN was in fact more evident in patients with moderate malnutrition (SGA stage B) than those with severe malnutrition (SGA stage C).

Thabet et al. [116] have shown that the IDPN, as already described for the ONS, improves the nutritional status of the patient, improves anemia status and therefore reduces the necessary dose of EPO therapy. The authors conducted a study on 40 HD patients with refractory anemia and malnutrition. The participants were divided into a control group and an experimental group under IDPN for a period of six months. The data obtained at the time of enrollment, after three and six months, showed a progressive and significant increase in albumin, BMI and especially hemoglobin. Moreover, the values of the malnutrition inflammation score (MIS) were reduced. The MIS [135] is a questionnaire used for the assessment of nutritional status that includes a part on the patient’s self-completed history about dietary intake and possible interdialytic symptoms, and a part compiled by the medical-nursing staff that reports on the physical examination, the value of BMI, albumin and transferrin. Transferrin is an indicator of depletion of protein deposits and therefore of nutritional status [136].

### 3.4. Enteral and Total Parenteral Nutrition

In patients with severe malnutrition and a spontaneous energy intake of less than 20 kcal/day, ONS and IDPN are generally unable to guarantee an adequate nutritional status. Under such conditions, as well as in the presence of impaired swallowing capacity, daily nutritional support is required. The therapeutic possibilities are EN and TPN. Generally, if possible, it is preferable to use EN than TPN [89]. According to the guidelines of ESPEN (European Society for Clinical Nutrition and Metabolism) [137], EN must be taken into account in severely malnourished patients with a BMI < 20 kg/m^2^, a reduction in body weight > 10% in the last 6 months, albumin values < 3.5 g/dL and pre-albumin values < 300 mg/L. Frequently, these patients also have other comorbidities (such as central or peripheral neurological diseases) that do not allow the administration of ONS or the achievement of an adequate nutritional status via this administration. EN can be administered via nasogastric or naso-jejunal in case of gastroparesis and non-responsibility to pro-kinetic agents, or through percutaneous endoscopic gastrostomy (PEG) [90]. EN is also less expensive compared to TPN, and at the same time presents a lower risk of infectious or metabolic complications. 

The enteral administration of nutrients has a trophic action towards the gastrointestinal mucosa, improving its integrity, damaged by the uremic toxins. Parenteral administration, in addition to requiring good vascular access which is not always available, can have an irritating effect on the vascular district itself [90]. Although the use of EN is preferable, the choice of administering TPN is mandatory in the case of severe gastrointestinal dysfunction such as intestinal ischemia, gastro-intestinal obstruction or peritonitis [89]. TPN is the last possible choice in the event of failure of adequate nutritional intake through ONS, EN and IDPN.

## 4. Other Special Types of Nutritional Supplements

### 4.1. Ω-3 PUFAs

Ω-3 polyunsaturated fatty acids (PUFAs) are known for their multiple protective effects against CV and inflammatory diseases [138]. Smith et al. [139] conducted a study on 16 elderly patients without a noteworthy pathology subdividing them into two subgroups (group 1 and group 2). For 8 weeks, group 1 was given maize oil at the dose of 4 g/day, while Group 2 was given ω-3 at the dose of 4 g/day, of which a share was represented respectively by 1.86 g of eicosapentaenoic acid (EPA) and by 1.50 g of docosahexaenoic acid (DHA). Authors noted that only in group 2 individuals who were given ω-3 was there an increase in the rate of muscle protein synthesis (MPS). The MPS monitors the dosage of phenylalanine and leucine, considered the main regulators of protein synthesis. An increase of the level of these two amino acids is therefore related to an enhancement in muscle protein synthesis and therefore of MPS. The results of the study also showed an increase in muscle expression of mammalian target of rapamycin (mTOR) and ribosomal protein S6 kinase beta-1 (p70S6K), two protein kinases involved in intracellular signaling, responsible at the muscular level for the activation of anabolic protein processes. Such data show that, in older patients, ω-3 PUFAs would stimulate muscle protein synthesis by countering catabolic events and sarcopenia. Studies of the role of ω-3 PUFAs in the prevention of sarcopenia in CKD patients are still few. Azemi et al. [118] conducted a study of 120 HD patients randomly divided into 4 subgroups (group 1, 2, 3, 4), for a total duration of 12 months. Only vitamin E was administered to group 1, only ω-3 PUFAs to group 2, ω-3 PUFAs and vitamin E together to group 3, and placebo to group 4. The administration of ω-3 PUFAs in groups 2 and 3 showed benefits in reducing the SGA score. Ω-3 PUFAs also reduced glucose plasma fasting, serum insulin levels, homeostasis model of assessment (HOMA)-IR, and improved the quantitative insulin sensitivity check index (QUICKI). The administration of ω-3 would allow an improvement in nutritional status by preventing the development of diseases such as sarcopenia, and at the same time would improve the glycol-metabolic status of the patient, reducing the status of IR. The latter is particularly important considering that diabetes is a common in CKD patients and is the cause of a further worsening of their prognosis [140].

Gharekhani et al. [117] conducted a study of 25 chronic HD patients divided into two groups, a control group and an experimental group, to which ω-3 PUFAs were administered daily for four months. The authors pointed out that, following the administration of ω-3 PUFAs, there was an increase in pre-albumin values and a reduction in ferritin, IL-10 and IL-6 (known inflammation indices). This study demonstrated the ability of ω-3 PUFAs to improve both the nutritional status of the patient and the systemic inflammatory state notoriously increased in CKD patients. Dessì et al. [141] conducted a study of 99 HD patients (experimental group) and 160 healthy subjects who did not take any ONS or drugs (control group). It was observed that chronic HD patients had a reduced intake of protein, carbohydrates and lipids and therefore a reduced total energy intake, confirming the increased risk of malnutrition present in this category of patients. Furthermore, in the experimental group, a reduction of PUFAs was observed at both plasma and erythrocytic membrane levels. Erythrocytes are used as model cells for assessing membrane fatty acid composition and phospholipid intake through diet. In particular, an increase in the ω-6/ω-3 ratio was observed, mainly due to the reduction of ω-3. The reduction of PUFAs and especially ω-3 was associated with an increased risk of CV events and mortality, already notoriously high in HD patients. The reduction in plasma and erythrocyte PUFA concentration appears to be due not only to reduced dietary intake, but also to the loss of such elements in dialysis during HD treatment [142,143]. This study confirms the importance of ensuring a nutritional diet that provides an adequate amount of the different nutrients, including ω-3. Such intervention would lead to an improvement in the prognosis of HD reducing the risk of malnutrition and especially of CV events and mortality [144].

### 4.2. Fiber

The use of fiber as a nutritional supplement has shown benefits in reducing toxins related to uremic proteins produced by the gut microbiota such as indoxyl sulfate and p-cresol. The plasma reduction of these toxins according to various studies would lead to an improvement in the prognosis of CKD patients reducing their mortality. Dysbiosis, in fact, through the production of uremic toxins, participates in the establishment of the systemic inflammatory status typical of CKD patients, responsible for catabolic protein processes at the muscular level that will lead to the development of sarcopenia [70]. Fiber ingestion is associated not only with the reduction of uremic toxins but also with the reduction of creatinine and azotemia values [145]. Krishnamurthy et al. [119] analyzed the data from the NAHNES III study [86] including 14,543 patients. The authors noted that in CKD patients (5.8% of the population analyzed) with a fiber intake of 10 g/day, the probability of detecting an increase in CRP changed from 11% to 38%. The mortality rate was also inversely related to the quantity of fiber intake. The ingestion of fibers would seem correlated to a reduction in the degree of chronic inflammation present in the CKD patient and a reduction of mortality. According to data from the NHANES III [145], it would appear that CKD patients take low amounts of fiber i.e., on average 15.4 g/day. The recommended intake of fiber in nephropathic patients is 25–30 g/day, as in the normal population. The reduced intake can be explained by dietary restrictions imposed due to potassium content, which is widely present in vegetables. For this reason, in CKD patients careful nutritional counselling should be performed to allow appropriate management of the dietary-nutritional plan. 

## 5. Other Therapeutic Approaches to Uremic Sarcopenia

Physical exercise was the most commonly studied and validated therapy for the treatment of both primary and secondary sarcopenia [39]. The performance of both aerobic and anaerobic physical exercises has been shown to reduce the degree of sarcopenia thanks to the progressive increase of MM and muscle strength. Physical exercise also allows an improvement in sarcopenia as it also leads to a reduction of IR and anorexia, processes involved in the reduction of MM [146]. In view of the generally sedentary life of HD patients, the beginning of a physical exercise program adapted to the age and comorbidity of the individual has demonstrated beneficial effects on both MM and strength and is recommended in all patients at risk for the development of sarcopenia. A further possible approach for the treatment and prevention of sarcopenia is to intervene in the processes involved in the alteration of the balance between catabolism and protein anabolism, the cause of sarcopenia.

Poor physical activity is one of the most frequent disabling problems in ESRD patients, confirmed by the fact that several studies have highlighted the benefits derived from physical exercise in hemodialysis patients [16,20,31]. These data were confirmed by a multi-center randomized clinical trial, “Exercise introduction to enhance performance in dialysis” [147] with six months follow-up which evaluated the effect of a simple walk-based exercise program, conducted at home, supervised by the staff of the dialysis center, on the functional status of hemodialysis patients. Specifically, 296 HD patients were divided into two subgroups, of which the first one represented the control group and performed normal physical activity, while the second group was instructed in the walking-based physical activity protocol. After six months of intervention, it appeared that the group subjected to walking exercise had an improvement in physical performance assessed by the six minutes walking test and the five times sit-to-stand test. In addition, physical activity also positively impacted on the cognitive function and on the quality of social interactions of patients. These parameters were monitored by the Kidney Disease Quality of Life Short Form questionnaire.

A recent study has highlighted how medium or high intensity intra-dialytic resistance exercise is a useful therapeutic tool in HD patients [148]. In fact, after 12 weeks of this exercise program, repeated three times a week, dialysis efficiency improved. Furthermore, physical exercise also seemed to have a positive impact on inflammatory parameters, improving the chronic inflammatory status of uremic patients.

A subsequent study compared high-intensity and moderate-intensity resistance exercise repeated for 12 weeks in a group of hemodialysis patients, demonstrating that high-intensity intradialytic exercise compared to the other form of exercise induced an enhancement in lower limb muscle mass and quality of life [149], while other parameters such as functional capacity, appendicular muscle mass and sarcopenia ameliorated regardless of the intensity of physical exercise.

The positive impact of physical activity was observed not only in HD patients but also in CKD patients under conservative therapy. Secondary analysis from the ExTra CKD study showed that supervised physical exercise in CKD patients under conservative therapy is able to significantly improve the volume of the quadriceps femoris [150]. Furthermore, studies conducted on CKD non-dialysis patients demonstrated that aerobic exercise, intermittent exercise, and resistance training are able to enhance VO_2_ peak [151,152,153]. 

The combination of nutritional therapy with physical exercise could represent a further useful tool to counteract the onset and progression of sarcopenia. In fact, as previously demonstrated by several studies, the two therapeutic approaches could be more effective in combination than the single treatment [154]. In fact, in conservative therapy for patients with CKD, the combination of LPD with endurance exercise counteracts the loss of muscle mass [155]. This study highlighted that endurance training carried out during LPD reduces protein catabolism, with an improvement in body composition. This is supported by the fact that a direct correlation was observed in patients undergoing this training program between the increase in type I and II muscle fibers, that of muscle strength, and serum albumin levels. This is also associated with a reduction in the inflammatory state. A study conducted on HD patients demonstrated that intra-dialytic parenteral nutrition, associated with physical exercise, was able to increase the absorption of essential amino acids following the dialysis session, compared to a group of HD patients treated with intra-dialytic parenteral nutrition alone. In particular, these authors observed an increase in the uptake of amino acids in the forearm [156].

In order to avoid a state of metabolic acidosis, it is desirable to maintain bicarbonate values above 22 mmol/L through a suitable HD treatment or administration of sodium bicarbonate. According to a study by Boirie et al., a concentration of 16 mmol/L bicarbonates would result in double protein muscle loss compared to values > 22 mmol/L [157]. IR is an important risk factor for the development of sarcopenia, and can be counteracted primarily through constant exercise, especially if aerobic [158]. Among the drugs used for diabetes mellitus (DM), thiazolidinediones have gained particular interest as they have demonstrated the ability to activate the PI3K pathway, generally suppressed in sarcopenia by the UPS system; further studies are required to validate the efficacy of thiazolidinediones in increasing MM in sarcopenic patients [159].

Hormonal therapy with nandrolone, due to reduced testosterone concentration in CKD patients, unfortunately has limited use due to possible side effects such as erectile dysfunction, gynecomastia and increased CV risk [160]. Among future therapeutic perspectives that are being studied with murine samples, we mention the use of microRNA and sirtuins (SIRTs). MicroRNAs, by binding specific regions of the 3′ end, named untranslated regions (UTRs), of the mRNA, block its translation and thus limit the expression of certain gene regions. The use of microRNAs could reduce the expression of mRNA areas involved in muscle protein degradation or reduced myogenesis [161,162]. The functional integrity of the mitochondria is fundamental for the metabolism of the whole cell and its alteration is associated with the loss of muscle tissue [163]. It has been observed that in HD patients there is a reduction of peroxisome gamma proliferator coactivator 1α (PGC-1α), a protein responsible for the expression of genes involved in the regulation of mitochondrial activity. A reduction of PGC-1α would cause impaired mitochondrial function [164]. SIRTs are enzymes responsible for the stimulation of mitochondrial biosynthesis, and their use in the expression of murine has been associated with slow aging and loss of MM [165].

## 6. Conclusions

Sarcopenia is defined by the reduction of MM, associated with the loss of muscle strength and the reduction of physical performance. It is a frequent condition in CKD patients, especially if undergoing HD treatment. The development of sarcopenia in nephropathic patients is not associated solely with their generally advanced age. Uremic sarcopenia is mainly related to the comorbidities typical of uremia: metabolic acidosis, low-grade chronic inflammatory state, vitamin D deficiency, IR, hormonal alterations (in particular testosterone, IGF-1 and cortisol) and gut dysbiosis. These processes contribute to the development of an increased protein catabolism and of a reduced protein synthesis, resulting in a reduction of the muscle protein pool that induces a loss of MM and strength. The same hemodialytic treatment, fundamental for the patient’s survival in ESRD, causes the loss of amino acids and proteins. During and after the HD session, an increase in the processes of protein catabolism was also observed. The development of sarcopenia causes a worsening of the prognosis of CKD patients, leading not only to a worsening of Qol and of personal autonomy, but also to an increased risk of complications and mortality.

For this reason, sarcopenia should be suspected and diagnosed as soon as possible in patients considered at risk. There are many tools available to investigate the presence of this disease, the choice of which is often dictated by the clinician and by diagnostic equipment availability. In reference to these latter, the most commonly used are DXA, BIA or HGS. Sarcopenia treatment is essential to improve the prognosis and survival of CKD patients. Physical exercise has been studied in several studies [154,166,167] and has provided encouraging results, but CKD patients cannot always perform it optimally, especially if they are very old and suffering from multiple comorbidities. Nutritional therapy could be a valid and effective solution in ensuring a correct protein and energy intake, which contrasts with the increased protein catabolism and the reduced protein synthesis.

The possible types of nutritional therapy administration are multiple and include ONS, EN, TPN and IDPN. The assumption of supplements such as fiber or ω-3 PUFAs could also be useful in counteracting the onset and progression of sarcopenia. Studies conducted so far on different types of nutritional therapies [114,115,123,133] have provided positive results, so their use, associated with careful nutritional counselling, should be encouraged in CKD patients.

Currently, it is believed that the combination of targeted and personalized physical exercise for the patient with personalized dietary-nutritional therapy [168] to ensure an adequate protein, fiber and energy intake represents the ideal approach to treat uremic sarcopenia.

## Figures and Tables

**Table 1:** Possible nutritional treatments of uremic sarcopenia in humans.

Nutritional Approaches	Author	Year	Study Population	Nutritional Treatment	Primary Outcome	Primary End-Point
**ONS**	Caglar K. et al. [110]	2002	85 malnourished CHD, HD patients	ONS assumed during each HD session, containing 16.6 g of proteins, 22.7 g of lipids and 52.8 g of carbohydrates with energy content of 475 kcal.	Significant increases in serum albumin and prealbumin levels were detected. In addition, there was a 14% increase in SGA score.	ONS assumed during HD improves some nutritional biomarkers in malnourished HD patients.
	Lacson Jr E. et al. [111]	2012	5.227 HD patients with albumin level ≤3.5 g/dL vs. 5.227 patients (control group)	Four different intradialytic ONS were administered: (a) 19 g proteins, 425 kcal for dose; (b) 15 g proteins, 60 kcal for dose; (c) 14 g proteins, 210 kcal for dose; (d) 20 g proteins, 210 kcal for dose.	A reduction in mortality was found in patients treated with ONS compared to non-treated group.	ONS treatment allows a significant increase in survival of HD patients.
	Wu H.L. et al. [84]	2013	55 CKD patients (stage III-IV) vs. 54 patients (control group)	One daily ONS containing 0.6 g of proteins, 8.2 g of lipids, 30.9 g of carbohydrates and 1.9 g of fiber with energy content of 200 kcal.	ONS significant decreased urine protein excretion therefore, daily protein intake was lower in the ONS group. Significant decrease of creatinine and urea nitrogen levels; in addition, there was a significant increase of eGFR.	ONS has improved some blood parameters and improved the adherence to the nutritional therapy with less protein excretion.
	Sezer S. et al. [112]	2014	32 malnourished HD patients vs. 30 patients (control group)	ONS containing 14 g of proteins, 19.2 g of lipids and 41.3 g of carbohydrates with energy content of 400 kcal. In addition, during HD sessions was served a snack containing 14 g of proteins, 10 g of lipids and 55 g of carbohydrates with energy content of 300 kcal.	Significant increases in serum albumin levels were detected. Furthermore, there was a significant increase in the dry weight of the ONS patients and a significative reduction in the dry weight of the control group. In addition, a reduction of EPO dose requirement and MIS was detected in the treated group.	ONS treatment improves serum albumin levels and allows a lower EPO dose requirement in HD patients.
	Benner D. et al. [113]	2018	3.374 HD patients with albumin level ≤3.5 g/dL vs. 3.374 patients (control group)	Two different ONS were used: (a) 21.6 g of proteins and 475 kcal for dose; (b) 16 g of proteins and 70 kcal for dose.	There was a 69% reduction in mortality and a 33% reduction in missed dialysis sessions.	ONS treatment allows a significant increase in survival in HD patients with albumin level ≤3.5 g/dL.
	Leonberg-Yoo A.K. et al. [114]	2019	1420 HD patients vs. 4.059 patients (control group)	Six different intradialytic ONS were used: (a) 19 g proteins and 425 kcal for dose; (b) 15 g proteins and 60 kcal for dose; (c) 14 g proteins and 210 kcal for dose; (d) 20 g proteins and 210 kcal for dose; (e) 16 g proteins and 90 kcal for dose; (f) 16 g proteins and 160 kcal for dose.	There was a decrease of re-hospitalization within 30 days of first discharge.	ONS treatment reduces post-discharge hospital readmission rates.
**IDPN**	Marsen T.A. et al. [115]	2017	39 HD patients with PEW vs. 44 patients (control group)	IDPN treatment three times/week containing (one dose): glucose (70%); amino acids (15%); lipids (20%) vitamins; L-carnitine.	Significant increases in serum prealbumin levels were detected.	IDPN used during HD session improves prealbumin levels.
	Thabet A.F. et al. [116]	2017	20 HD patients vs. 20 patients (control group)	IDPN treatment three times/week. In addition, patients received EPO, iron dextran, folic acid and vitamin B 12.	Significant increases in hemoglobin and albumin levels were detected. In addition, there was a significant increase in BMI. Significant reduction in MIS was detected.	IDPN treatment allows an improvement of refractory anemia, as it permits an increase in hemoglobin and prealbumin levels and also an increase in body weight. It also leads to a reduction in MIS.
	Deleaval P. et al. [60]	2020	6 HD patients	Two dialysates were used during HD treatment: Standard dyalisate; Dialysate enriched in BCAA (valine, isoleucine, leucine).	During the HD treatment with standard dialysate a reduction in plasmatic valine was found, while with dialysate enriched in BCAA HD treatment there was an increase in plasmatic valine, isoleucine and leucine.	The use of dialysate enriched in BCAA allows the restoration of normal plasma BCAA levels.
**ω-3 supplementation**	Gharekhani A. et al. [117]	2014	27 HD patients vs. 27 patients (control group)	Six capsules *per* day of ω-3 supplementation (180 mg eicosapentaenoic acid and 120 mg docosahexaenoic acid in each capsule).	ω-3 supplementation is a significant independent predictor for the increase of serum prealbumin level after adjusting post-treatment nutritional markers. Significant decrease in ferritin levels and IL-10/IL-6 ratio was detected.	ω-3 supplementation in HD patients permits a slight reduction of inflammation.
	Asemi Z. et al. [118]	2016	90 HD patients vs. 30 patients (control group)	Four groups for supplementation *per* day: 1250 mg/day ω-3 PUFA containing 600 mg eicosapentaenoic acid and 300 mg docosahexaenoic acid; 400 IU/day vitamin E; 1250 mg ω-3 PUFA containing 600 mg eicosapentaenoic acid and 300 mg docosahexaenoic acid + 400 IU vitamin E; placebo (control group).	Significant reduction in SGA, FPG, insulin levels and HOMA-IR were detected. In addition, there was a significant enhancement in QUICKI.	ω-3 PUFA and vitamin E combined supplementation improve SGA and the metabolic profile in HD patients.
**Fiber**	Krishnamurthy V.M.R. et al. [119]	2012	1.105 CKD patients (stage IIIa-IV) vs. 13.438 subjects (control group)	Two groups were divided into two subgroups according to fiber dietary intake: Low total fiber (<14.5 g/day); High total fiber (≥14.6 g/day).	Significant decrease in CRP was detected in CKD patients with high total fiber dietary consumption.	The high dietary fiber consumption is associated with a minor inflammation risk and mortality in CKD patients.

Abbreviations: BCAA, Branched-chain amino acid; BIA, Bioelectrical impedance analysis; BMI, Body mass index; CHD, Coronary heart disease; CKD, Chronic kidney disease; CRP, C-reactive protein; e-GFR, Estimated glomerular filtration rate; EPO, Erythropoietin; FPG, Fasting plasma glucose; HD, Hemodialysis; HOMA-IR, Homeostasis model of assessment of insulin resistance; IDPN, Intra-dialytic parenteral nutrition; IL, Interleukin; MIS, Malnutrition inflammation score; MPS, Muscle protein synthesis; ONS, Oral nutritional supplements; PUFA, Polyunsaturated fatty acids; QUICKI, Quantitative insulin sensitivity check index; SGA, Subjective global assessment.

## Abbreviations

BIA	Bioimpedance analysis
BMI	Body mass index
b.w.	Body weight
CKD	Chronic kidney disease
CPI	Controlled protein intake
CRP	C-reactive protein
CT	Computed tomography
CV	Cardiovascular
DHA	Docosahexaenoic acid
DM	Diabetes mellitus
DXA	Dual-energy X-ray absorptiometry
E3s	Ubiquitin ligases enzyme
EN	Enteral nutrition
EPA	Eicosapentaenoic acid
EPO	Erythropoietin
ESPEN	European society for clinical nutrition and metabolism
ESRD	End-stage renal disease
EWGSOP	European working group for sarcopenia for older people
FGF-23	Fibroblast growth factor-23
FoxO3A	Forkhead box O3A
GH	Growth hormone
GFR	Glomerular filtration rate
HD	Hemodialysis
HGS	Handgrip strength
HOMA	Homeostasis model of assessment
HPD	High-protein diet
IDPN	Intra-parenteral nutrition dialysis
IGF1	Insulin-like growth factor-1
IL	Interleukin
IR	Insulin resistance
ISRNM	International society of renal nutrition and metabolism
KAs	Ketoanalogues
LPD	Low protein diet
MAMC	Mid arm muscle circumference
MIS	Malnutrition inflammation score
MM	Muscle mass
MPS	Muscle protein synthesis
MRI	Magnetic resonance imaging
mTOR	Mammalian target of rapamycin
NHANES	National health and nutrition examination survey
ONS	Oral nutritional supplements
p70S6K	Ribosomal protein S6 kinase beta-1
PEG	Percutaneous endoscopic gastroctomy
PEW	Protein-energy wasting
PGC-1α	Peroxisome gamma proliferator coactivator-1α
PI3K	Protein kinase B
PUFAs	Polyunsatured fatty acids
Qol	Quality of life
QUICKI	Improved quantitative insulin sensitivity check index
SCWD	Society on sarcopenia, cachexia and wasting disorders
SGA	Subject global assessment
SIRTs	Sirtuins
SOCS-3	Suppressor of cytokine signaling-3
SPPB	Short physical performance battery
Stat3	Signal transducer and transcriptional activator 3
TNF-α	Tumor necrosis factor-α
TPN	Total parenteral nutrition
UPS	Ubiquitin proteasome system
UTRs	Untranslated regions
